# Bisphenol-A Leaching from Polycarbonate 5-Gallon Water Bottles in the UAE: A Comprehensive Study

**DOI:** 10.3126/nje.v14i1.59934

**Published:** 2024-06-30

**Authors:** Zahraa Khalid Ahmed Al-Tameemi, Razia Khanam, Preetha J Shetty

**Affiliations:** 1 Department of Biomedical Sciences, College of Medicine, Gulf Medical University, Ajman, UAE

**Keywords:** Bisphenol-A, Polycarbonate plastics, leaching, liquid-liquid extraction, sunlight

## Abstract

Bisphenol A (BPA) is widely used around the world in the production of Polycarbonate (PC) plastics. Notably, the ubiquitous 5-gallon water bottles in the UAE are primarily made of PC plastic, making them a significant concern as bottled water is the region’s main supply of drinking water. These bottles undergo temperature variations during storage and transportation, potentially leading to harmful BPA (Bisphenol A) leaching.

This study analyzed 40 PC 5-gallon water bottles from two local brands A and B, with 20 bottles per brand, under two conditions: room temperature and outdoor sunlight exposure for a month. BPA levels were assessed at 0, 15, and 30 days, following ethical approval. Liquid-liquid extraction and ELISA assays were conducted, with comprehensive kit validation.

The results revealed a significant increase in BPA concentration over time, particularly in bottles exposed to elevated temperatures (Day 30 outdoor-stored samples exhibited the highest concentration at 9.05 ± 2.30 μg/L). Brand B consistently exhibited higher BPA concentrations across different samples and environments.

This study emphasizes the link between BPA content and storage time, highlighting the need for preventive measures to reduce BPA exposure. Individuals should be aware of potential health risks associated with prolonged storage in plastic containers and consider safer alternatives.

## Introduction

Bisphenol A (BPA) is a ubiquitous plastic monomer and plasticizer, ranking among the most produced chemicals worldwide, with an annual output surpassing 6 million pounds [1-2]. It is a fundamental component in the making of variety of products, including polycarbonate plastics, coatings used in lining metal cans, and a wide array of consumer items such as toys, water pipes, eyeglass lenses, and medical equipment. Its pervasive use is attributed to its diverse physicochemical properties and versatility in a multitude of applications [3].

The tendency of BPA to seep into composites, dental sealants, and food and beverage containers, even under routine conditions of use has raised concerns. Extensive studies have shown that BPA migrates into food and beverages that come into contact with polycarbonate containers [4-6]. This migration is a complex process influenced by various factors, including photolytic formation and degradation of organic compounds, thus underscoring the importance of understanding these mechanisms.

The widespread occurrence of BPA in various human biological fluids such as serum, urine, amniotic fluid, placental tissue, and umbilical cord blood suggests the likelihood of human exposure [7-8]. Importantly, in certain instances, the levels of total BPA, including both free and conjugated forms, in human biological fluids exceed concentrations known to trigger specific molecular responses in animal studies. This prompts concerns about the potential health effects of such exposure, especially within the framework of “low doses” of endocrine-disrupting chemicals, as outlined by the NIEHS Low Dose Peer Review [9].

While BPA has traditionally been identified as having weaker estrogenic effects compared to natural estrogens such as estradiol, recent studies have revealed that BPA can trigger cellular responses at extremely low concentrations through various pathways [10]. This includes its interaction with estrogen receptors (ER), with an approximately tenfold higher affinity for ERβ than ERα [11-14]. This revelation challenges previous assumptions and underscores the necessity of comprehending the multifaceted mechanisms through which BPA can impact cellular responses.

The widespread availability of BPA in the environment, coupled with its estrogenic effects in certain in vitro and in vivo responses, raises worries about potential negative health impacts [15]. Notably, early-life exposure to BPA may contribute significantly to the onset of various health issues, such as infertility, abnormalities in genital tract abnormalities, obesity, attention deficit hyperactivity disorder (ADHD), and prostate and breast cancer [16]. Human exposure mainly happens through ingestion, although absorption through the skin from bathing in BPA-contaminated and inhalation are also possible routes.

In regions such as the United Arab Emirates (UAE), where bottled water serves as the primary source of drinking water, the potential consequences of BPA leaching from plastic water bottles are of relevance. These bottles are subjected to varying temperatures during delivery and storage, which could impact the levels of BPA leaching and, consequently, pose health risks. Therefore, the need to detect and quantify BPA levels in bottled water becomes crucial to determine best practices for minimizing BPA leaching.

Existing research has not adequately addressed the influence of temperature and storage conditions on BPA leaching from plastic water bottles, particularly in areas where bottled water is the main source of drinking water. This lack of information highlights the significance of the current study. The objective of this study is to quantify the levels of BPA leaching from 5-gallon drinking water bottles when stored under room temperature and outdoor sunlight exposure conditions and for varying durations and to establish appropriate precautions and recommendations to minimize BPA leaching from commercial water bottles, contributing to safer drinking water practices

## Methodology

### Study Site and Ethical Clearance

This investigation was executed at the Thumbay Labs within the premises of Gulf Medical University, located in Ajman, UAE. The research aimed to assess the concentration of Bisphenol A (BPA) in 5-gallon bottled water originating from two distinct brands, both acquired from the local UAE market. Prior to commencing the study, ethical approval was granted by the Ethics Committee of Gulf Medical University in accordance with ethical standards.

### Sample Collection

**Figure 1:**
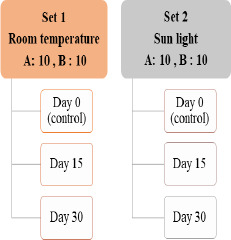
Sample Setting

A total of 40 samples were included in this study. Two distinct brands, each consisting of 20 samples, were selected, and purchased from local markets in the UAE. For each brand, two sets were established: Set 1 was maintained indoors at a constant room temperature of 23°C, while Set 2 was placed outdoors under direct sunlight, with an average daily temperature exceeding 44°C. BPA concentration measurements were taken from all samples on three separate occasions: day 0, day 15, and day 30 (Figure 1).

The methodology encompassed two key stages: sample preparation using the liquid-liquid extraction method, and quantitative detection through enzyme-linked immunosorbent assay (ELISA).

Test Validation: To ensure the reliability of the ELISA kit, several validation tests were conducted.

- Preliminary Test: A preliminary examination was performed as specified by the kit manufacturer. The results from this test aligned well within the kit's specifications, as illustrated in Table 1.
- Linearity Test: The linearity of the kit was verified by assessing absorbance at five different concentrations, and the results demonstrated an R2 value consistent with the kit's specifications, as documented in Table 2.
- Specificity Test: The kit's specificity was assessed by determining the decrease in transmission as BPA concentration increased (shown as B/B0), as recorded in Table 3.

Extraction of Bisphenol-A: The extraction of BPA was conducted using the liquid-liquid extraction method with chloroform as the organic solvent. This technique is established on the separation of BPA based on its differential solubility in two immiscible liquids, namely water and chloroform. A separating funnel was employed for the separation of the organic layer.

Sample Preparation: The sample preparation process involved the following steps: 100 ml of the sample was transferred to a separating funnel, and two drops of 0.1 M HCl were added. Subsequently, 50 ml of chloroform was added to the sample, and the mixture was agitated for 25 minutes. Following agitation, the organic layer was meticulously separated into a beaker and allowed to dry. Once the chloroform had completely evaporated, 1-2 ml of methanol was added to the dried residue. Finally, the prepared sample was transferred to a test tube, rendering it ready for analysis.

Estimation of Bisphenol-A: The estimation of Bisphenol A in water samples was accomplished using the BPA ELISA kit, catalog number KA1495 from ABNOVA, Taiwan. This procedure was executed in duplicate, strictly adhering to the protocol stipulated in the kit.

### Statistics

All estimated values were presented as Mean ± Standard deviation. The concentration of BPA was compared between control and test groups using Student’s t Unpaired Test (p<0.05).

## Results

The primary objective of this study was to evaluate the concentration of Bisphenol-A (BPA) in freshly filled drinking water stored in 5-gallon polycarbonate plastic bottles. The analysis involved 40 water samples, which included two different brands, with 20 samples from each brand. These samples were assessed under varying conditions, including storage at room temperature and exposure to direct sunlight for three time points (day 0, day 15, and day 30).

Effect of Storage Conditions on BPA Concentration: Comparison of the mean BPA concentrations revealed notable differences influenced by both the duration of storage and environmental conditions (Table 4). At the start of the study (day 0), no significant difference was observed in mean BPA concentrations between the two sets, which were water samples stored at room temperature and those exposed to sunlight. However, by day 15, a substantial increase was noted in the mean BPA concentration of water samples exposed to sunlight (4.01 ± 0.73 μg/L) in contrast to those stored at room temperature (0.80 ± 0.17 μg/L), with a statistically significant difference (p value < 0.001). On day 30, this trend persisted, showing an even greater disparity in BPA concentration, with the mean for sunlight-exposed samples being 9.05 ± 2.30 μg/L and room temperature samples having a mean concentration of 2.31 ± 0.16 μg/L (p value < 0.001).

Comparison between Brands: The study also considered differences in BPA concentration between brands (A and B) when samples were stored at room temperature. At the study's commencement (day 0), the mean BPA concentrations for brand A and brand B were 0.37 ± 0.24 μg/L and 0.36 ± 0.16 μg/L, respectively. These initial measurements suggested no significant difference in BPA concentration between the two brands. However, by day 15, a significant difference emerged, with brand A having a lower mean concentration of 0.67 ± 0.05 μg/L compared to brand B (0.94 ± 0.12 μg/L). This discrepancy persisted on day 30, with brand A and brand B exhibiting mean concentrations of 2.16 μg/L and 2.45 μg/L, respectively (p value < 0.001) (Table 5).

Additionally, when the concentration of BPA in water samples from brands A and B was compared under sunlight exposure, brand B consistently exhibited significantly higher BPA concentrations than brand A (Table 6). On day 15, brand B showed a mean concentration of 4.68 μg/L, while brand A had a mean of 3.33 μg/L (p value < 0.001). This trend continued on day 30, with brand B registering a mean concentration of 11.27 μg/L, whereas brand A had a mean concentration of 6.83 μg/L (p value < 0.001).

The study results indicate that BPA concentrations are significantly affected by both storage duration and environmental factors, with increased exposure to sunlight leading to higher BPA concentrations. Furthermore, differences in BPA concentration between the two brands suggest that the type of polycarbonate plastic used in the bottles may play a significant role in BPA leaching.

## Discussion

The majority of research has primarily concentrated on BPA exposure originating from dietary sources, with particular attention given to assessing BPA levels in food, particularly items stored in cans with epoxy resin linings. However, alternative avenues of BPA exposure, including drinking water, air, and dust, have received relatively less scrutiny in scientific investigations.

In the current study, the primary focus centers on estimating BPA concentrations in bottled drinking water, revealing a concentration of approximately 0.36 ± 0.02 μg/L in newly filled 5-gallon bottles. It has been established that BPA can migrate in minute quantities into food and beverages that come into contact with polycarbonate (PC) containers [17]. Leaching processes, such as photolytic formation and organic compound degradation, contribute to BPA migration during storage, as affirmed by several studies, which indicate that BPA leaches from polycarbonate plastics upon contact with their contents [18-20].

The United States Food and Drug Administration (USFDA) determined a no-observed-adverse-effect level (NOAEL) for BPA at 5 mg/kg/day based on the findings of two multigenerational rodent studies [21]. In contrast, the European Food Safety Authority (EFSA) set a significantly lower total tolerable daily intake (TDI) of 0.05 mg BPA/kg body weight in 2015 [22]. Furthermore, the oral reference dose was established at 100 μg/L as a total allowable concentration (TAC). The EFSA has specified a temporary tolerable daily intake (t-TDI) for BPA at 4 μg/kg body weight/day [23].

This study highlights that BPA concentrations in stored water increase with the duration of storage, possibly due to leaching from the containers resulting from photolytic breakdown or organic compound degradation during storage [20,24]. Earlier investigations conducted in Germany aimed at assessing potential risks to the general population have detected BPA in river and drinking water samples at varying concentrations [25]. Furthermore, a comprehensive study of wastewater contaminants revealed detectable BPA levels in 41.2% of 139 streams sampled across 30 U.S. states [26].

The study demonstrates that BPA concentrations rise when water is stored outdoors at elevated temperatures compared to water bottles stored at room temperature, indicating leaching from polycarbonate bottles under higher temperatures. This aligns with the findings of Baz et al., and Elobeid et al., who observed significant differences between bottles stored at different temperatures [19,27]. BPA leaching has also been observed in polycarbonate baby bottles from various countries [18], although the effects of washing, boiling, and brushing on BPA leaching have yielded diverse results.

Comparing two common brands used in Ajman, it was found that brand A exhibited lower BPA concentrations on the 15th and 30th days of storage compared to brand B. This discrepancy may be attributed to variations in bottle quality stemming from differences in raw materials and production technologies [24].

The current acceptable level of bisphenol A set by the U.S. Environmental Protection Agency is 50 micrograms/kg body weight/day [28-29]. However, this study reveals that the maximum BPA concentration in freshly filled bottles stored in sunlight for 30 days was 11.75 μg/L. For an average adult with a weight of 70 kg consuming 2 liters of water daily, this would result in an intake of 23.25 micrograms of BPA from water alone, which remains considerably lower than the recommended Tolerable Daily Intake (TDI) for BPA.

Several animal studies have demonstrated that even extremely low concentrations of bisphenol A in the parts per trillion range can impact cell function, underscoring its potent effects [30-33].

As far as we know, this is the initial investigation in the UAE to evaluate BPA levels in drinking water under different storage durations and at increased temperatures. The use of an ELISA kit for BPA estimation provides a cost-effective and suitable method for small-scale laboratories, potentially raising awareness among users. This study, while valuable, does have few limitations such as the challenge of controlling variable outdoor temperatures. We focused on a comparative analysis of just two brands, although there is a multitude of brands accessible in the local market. Additionally, our assessment of leaching was limited to only one-month period, whereas its crucial to note that bottles typically have an expiration of three months from the time of filling.

Based on our findings, we recommend that to minimize potential health risks, it is advisable to store drinking water away from direct sunlight and for limited durations. Furthermore, we recommend prioritizing the use glass or steel containers for water storage over polycarbonate bottles.

## Conclusion

This study emphasizes the critical relationship between bisphenol A content and storage conditions of PC particularly in the context of 5-gallon water bottles commonly used in the UAE. By analyzing the 40 PC bottles from two local brands at room temperature and sunlight, this research explains the concerning trend of BPA leaching, especially with prolonged exposure to elevated temperature. Therefore, it is imperative for consumers to be cognizant of the risks associated with prolonged storage of drinking water in PC water containers. Considering the reliance on bottled water as a primary source of drinking water in the region, the study highlights the necessity for informed choices and the exploration of safer alternatives.

We are happy to share the brand names with public health officials in UAE. More generally, by raising awareness and advocating for regulatory measures, we can work towards minimizing BPA exposure and safeguarding public health in the UAE.

## Figures and Tables

**Table 1:** Preliminary Test Report

SL. No	Parameter	Kit specification (mean absorbance)	Experimental Observation (mean absorbance)	Remarks
**1**	Blank	< 0.050	0.02	Pass
**2**	Negative control	> 1.000	1.644	Pass
**3**	Standard 5	> 0.050	0.181	Pass

**Table 2:** Linearity Test

Concentration	Absorbance (Kit)	Absorbance (Observed)
**0**	1.639	1.644
**0.01**	1.579	1.578
**0.05**	1.78	1.167
**0.2**	0.764	0.756
**1**	0.331	0.328
**5**	0.169	0.181

**Table 3:** Specificity Test

SL. No	Standard	Conc. μg/ml	Mean Absorbance	B/B0
**1**	**Blank**	--	0.02	--
**2**	**Negative Control**	0	1.644	100%
**3**	**Standard 2**	0.01	1.167	96%
**4**	**Standard 5**	0.05	0.181	87%
**5**	**Sample 1**	0.014	1.511	92%
**6**	**Sample 2**	0.041	1.238	75%
**7**	**Sample 3**	0.061	1.12	68%
**8**	**Sample 4**	0.136	0.856	52%

**Table 4:** BPA concentration on Day 0, 15, 30 at room temperature and sun light

Days	Room Temp. Mean Conc. μg /L (n-20)	Sun Light Mean Conc. μg /L (n-20)	P value
**Day 0**	0.36 ± 0.02	0.36 ± 0.03	NS
**Day 15**	0.80 ± 0.17	4.01 ± 0.73	<0.001*
**Day 30**	2.31 ± 0.16	9.05 ± 2.30	<0.001*

The values are presented as Mean ± Standard deviation

n- Number of samples,

* Statistically significant

**Table 5:** BPA concentration in water from brand A and B at room temperature

Days	Brand (n-10)	Mean Conc. μg /L	P value
**Day 0**	A	0.37 ± 0.24	NS
**Day 0**	B	0.36 ± 0.16	NS
**Day 15**	A	0.67 ± 0.05	<0.001*
**Day 15**	B	0.94 ± 0.12	<0.001*
**Day 30**	A	2.16 ± 0.07	<0.001*
**Day 30**	B	2.45 ± 0.07	<0.001*

The values are presented as Mean ± Standard deviation

n- Number of samples,

* Statistically significant

**Table 6:** BPA concentration in water from brands A and B in sunlight

Days	Brand (n-10)	Mean conc. μg /L	P value
**Day 0**	A	0.37 ± 0.02	NS
**Day 0**	B	0.36 ± 0.03	NS
**Day 15**	A	3.33 ± 0.31	<0.001*
**Day 15**	B	4.68 ± 0.06	<0.001*
**Day 30**	A	6.83 ± 0.13	<0.001*
**Day 30**	B	11.27 ± 0.43	<0.001*

The values are presented as Mean ± Standard deviation

n- Number of samples,

* Statistically significant
